# Supplementary material for: Identifying Risk Factors for Hospitalization with Behavioral Health Disorders and Concurrent Temperature-Related Illness in New York State
Source: Int J Environ Res Public Health. 2022 Dec 7;19(24):16411. doi: 10.3390/ijerph192416411 (PMC9779268; doi:10.3390/ijerph192416411)
Supplement: Supplementary file 1 [file ijerph-19-16411-s001.zip › ijerph-2039302-supplementary.pdf]

Supplementary Document

Identifying risk factors for hospitalization with behavioral health disorders and concurrent temperature-related illness in New York State

Heather Aydin-Ghormoz [1,2], Temilayo Adeyeye [1,2,*], Neil Muscatiello [1], Seema Nayak [1], Sanghamitra Savadatti [1,2] and Tabassum Z. Insaf [1,2]

[1] Center for Environmental Health, New York State Department of Health, Albany, NY 12208, USA
[2] School of Public Health, University at Albany, Rensselaer, NY 12144, USA
* Correspondence: temilayo.adeyeye@health.ny.gov

Table S1. Behavioral Health Disorders Diagnoses ICD-9/ICD-10 Crosswalk[1]

Diagnosis	ICD-10	ICD-9
Dementia	"F0390" "F05" "F0150" "F0151" "F062" "F060" "F0630" "F064" "F061" "F53" "F068" "F04" "F0280" "F0281" "F0390" "F0391"	"290" "293" "294"
Depressive Disorders	"F34" "F32" "F33" "F530" "N943" "F1014" "F1024" "F1094" F1114" F1124" "F1194" "F1314" "F1324" "F1394" "F1414" "F1424" "F1494" "F1514" "F1524" "F1594" "F1614" "F1624" "F1694" "F1814" "F1824" "F1894" "F1914" "F1924" "F1994" "F063" "F39"	"30110" "30113" "3004" "30112" "29699" "29621" "29622" "29623" "29624" "2980" "29625" "29626" "29682" "29620" "311" "29631" "29632" "29633" "29634" "29630" "29635" "29636" "29699" "29630" "6254" "29189" "2919" "29284" "30400" "30410" "30420" "30440" "30450" "30460" "29383" "29384" "2948" "29381" "29690"
Schizophrenia Spectrum and Other Psychotic Disorders	"F23" "F22" "F25" "F20" "F21" "F1015" "F1025" "F1095" "F1115" "F1125" "F1195" "F1215" "F1225" "F1295" "F1315" "F1325" "F1395" "F1415" "F1425" "F1495" "F1515" "F1525" "F1595" "F1615" "F1625" "F1695" "F1815" "F1825" "F1895" "F1915" "F1925" "F1995" "F060" "F061" "F062" "F24" "F28" "F29" "F53" "F531"	"2983" "2984" "2988" "2970" "2971" "2972" "295" "30122" "2915" "2913" "29189" "29211" "29212" "29289" "30400" "30430" "30410" "30420" "30440" "30450" "30460" "29381" "29382" "29389" "2948" "2973" "2989"
Schizophrenia	"F20" "F259"	"295"
Bipolar and Related Disorders	"F30" "F31" "F340" "F0633" "F0634"	"2960" "29681" "2964" "2965" "2966" "2967" "29689" "29680" "30110" "30113" "29383"
Anxiety Disorders	"F41" "F40" "F930" "F10180" "F10280" "F10980" "F12180" "F12280" "F12980" "F13180" F13280" "F13980" "F14180" "F14280" "F14980" "F15180" "F15280" "F15980" "F16180" "F16280" "F16980" "F18180" "F18280" "F18980" "F19180" "F19280" "F19980" "F064"	"3000" "3002" "30921" "29189" "29289" 30430" "30410" "30420" "30440" "30450" "30460" "29384"
Trauma- and Stressor-Related Disorders	"F43" "F942" "F941"	"3089" "30981" "3099" "3090" "3091" "30924" "30928" "3093" "3094" "30929" "30989" "3099" "31389"
Attention Deficit Hyperactivity Disorder	"F90"	"314"
Disruptive, Impulse-Control, and Conduct Disorders	"F602" "F91" "F63"	"3017" "3079" "3128" "31200" "31220" "3123" "31381" "31210"
Personality Disorder	"F21" "F07" "F60" "F688" "F69"	"3012" "3100" "3101" "3102" "31089" "3109" "3010" "3017" "3013" "30150"

		"30159" "3014" "3016" "3018" "30111" "3019" "30019"
Obsessive-Compulsive and Related Disorders	"F4522" "L981" "F42" "F633"	"3007" "6984" "3003" "3079" "31239"
Substance-Use Disorders*	"F10" "F12" "F14" "F16" "F18" "F11" "F19 "F15" "F630" "F13" "F17" "F55"	"291" "292" "303" "304" "305" "31231"
Alcohol Dependence	"F10229" "F1020" "F1021"	"303"
Drug Dependence	"F1120" "F1121" "F1320" "F1321" "F1420" "F1421" "F1220" "F1221" "F1520" "F1521" "F1620" "F1621" "F1920" "F1921"	"304"
Non-dependent Drug or Alcohol Use	"F1010" "F17200" "F1210" "F1290" "F1610" "F1310" "F1110" "F1410" "F1510" "F1910" "F1810"	"305"

[1] Includes depressive disorders, schizophrenia and other psychotic disorders, bipolar and related disorders, anxiety disorders, trauma and stressor related disorders, attention deficit hyperactivity disorders, disruptive, impulse control, and conduct disorders, personality disorders, and obsessive compulsive and related disorders. Excludes dementia since it is not a mental disorder.

Table S2. Temperature Related Illness Comorbidity ICD9/ ICD10 Crosswalk

Comorbidity	ICD-10	ICD-9
Diabetes	"E08" "E09" "E10" "E11" "E12" "E13"	"250"
Cardiovascular Disease	"I11" "I13" "I20" "I21" "I22" "I23" "I24" "I25" "I61" "I62" I63" "I64"	"402" "40401" "40411" "40491" "40403" "40413" "40493" "410" "411" "412" "413" "414" "42979" "4292" "431" "432" "43321" "43311" "43391" "43301" "43331" "43381" "43401" "43411" "43491"
Respiratory Disease	"J00" J01" "J02" "J03" "J04" "J05" "J06" "J09" J10" J11" J12" "J13" "J14" J15" "J16" "J17" "J18" "J20" "J21" "J22" "J30" "J31" "J32" "J33" "J34" "J35" "J36" "J37" "J38" "J39" "J40" "J41" "J42" "J43" "J44" "J45" "J46" "J47" "J60" "J61" "J62" "J63" "J64" "J65" "J66" "J67" "J68" "J69" "J70" "J80" J81" "J82" "J83" "J84" "J85" "J86" "J90" "J91" "J92" "J93" "J94" "J95" "J96" "J97" "J98"	"460" "461" "462" "463" "464" "465" "466" "471" "472" "473" "474" "475" "476" "477" "478" "480" "481" "482" "483" "484" "485" "486" "487" "488" "490" "491" "492" "493" "494" "495" "496" "500" "501" "502" "503" "504" "505" "506" "507" "508" "510" "511" "512" "513" "514" "515" "516" "517" "518" 519"
Obesity	"E66"	"278"

Table S3. Distribution of select climate parameters (2005 – 2019) in New York State

Climate Parameters[1]	Warm Months[2]			Cold Months[3]		
	Mean	Minimum	Maximum	Mean	Minimum	Maximum
Maximum Temperature (°C)	24.72	-0.67	39.21	7.99	-25.06	31.63
Maximum Heat Index (°C)	25.53	-0.67	50.28	7.46	-25.06	34.67

[1] Data extracted from the NASA North American Land Data Assimilation System (NLDAS), https://ldas.gsfc.nasa.gov/data , accessed on 28 September 2022

[2] May through September

[3] October through April

Table S4. Number of BHD Diagnoses Per Patient

BHD Diagnoses	Frequency	Percent	Cumulative Frequency	Cumulative Percent
**0**	223,734	1.82	223,734	1.82
**1**	7,585,768	61.81	7,809,502	63.63
**2**	2,703,433	22.03	10,512,935	85.66
**3**	1,298,933	10.58	11,811,868	96.25
**4**	349,962	2.85	12,161,830	99.10
**5**	85,109	0.69	12,246,939	99.79
**6**	20,742	0.17	12,267,681	99.96
**7**	4,171	0.03	12,271,852,	100.00
**8**	493	0.00	12,272,345	100.00
**9**	47	0.00	12,272,392	100.00
**10**	1	0.00	12,272,393	100.00

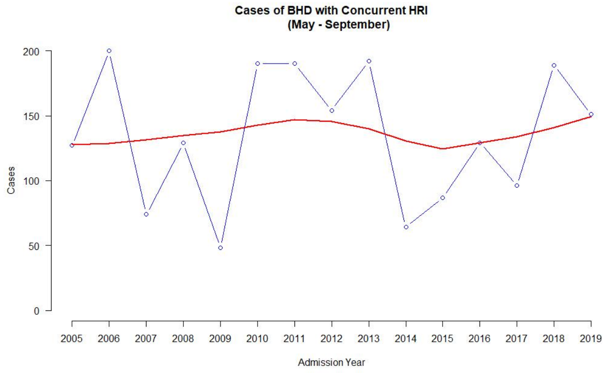

(**a**)

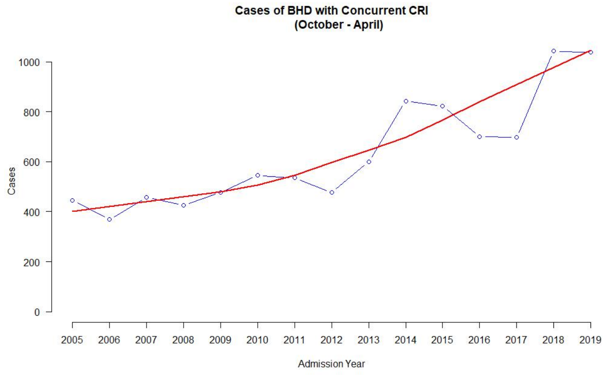

(**b**)

Figure S1. (**a**) Trends in Behavioral Health Disorders with Concurrent Heat-Related Illness; (**b**) Trends in Behavioral Health Disorders with Concurrent Cold-Related Illness.

Table S5. Hospitalization counts and risk ratios (RRs) of hospitalizations due to multiple primary or secondary diagnosis of behavioral health disorders (BHD) with a concurrent heat-related illness (HRI) (May-September) or cold-related illness (CRI) (October-April), 2005 – 2019

**BHD**[1]	**HRI**[2]				**CRI**[3]			
**Description**	**Count**	**%**	**RR**[4]	**95% CI**	**Count**	**%**	**RR**[4]	**95% CI**
**Behavioral Health Disorder Diagnoses**	**1,914**	**100**	-	-	**7,738**	**100**	-	-
Dementia and Schizophrenia	12	0.63	1.18	(0.67, 1.08)	60	0.78	1.45	(0.67, 1.87)
Dementia and Substance-Use Disorders	28	1.46	1.41	(0.97, 2.05)	204	2.58	**2.58**	**(2.24, 2.96)**
Schizophrenia and Substance-Use Disorders	66	3.45	1.04	(0.82, 1.33)	392	5.07	**1.60**	**(1.44, 1.77)**

[1] ICD codes: See Crosswalk in Table S1.

[2] Heat-Related Illnesses (HRI) ICD-9 992, E900.0, E900.9; ICD-10, T67, X30, X32

[3] Cold-Related Illnesses (CRI) ICD-9 991, E901.0, E901.9, E988.3; ICD-10 T68, T69, X31, T33, T34

[4] Risk ratios (relative risk) compared hospitalizations for BHD with concurrent TRI to hospitalizations for BHD without TRI, 2005 – 2019.

Table S6. Total Inpatient Hospitalizations in New York State (2005 – 2019)

Year	Count
**2005**	2,655,121
**2006**	2,669,814
**2007**	2,654,124
**2008**	2,648,018
**2009**	2,665,472
**2010**	2,622,277
**2011**	2,589,536
**2012**	2,544,866
**2013**	2,428,916
**2014**	2,367,984
**2015**	2,347,913
**2016**	2,340,901
**2017**	2,327,227
**2018**	2,352,842
**2019**	2,339,916
